# Updates on Functional Characterization of Bronchopulmonary Dysplasia – The Contribution of Lung Function Testing

**DOI:** 10.3389/fmed.2015.00035

**Published:** 2015-05-29

**Authors:** Anne Greenough, Anoop Pahuja

**Affiliations:** ^1^Division of Asthma, Allergy and Lung Biology, MRC and Asthma UK Centre in Allergic Mechanisms of Asthma, King’s College London, London, UK; ^2^NIHR Biomedical Research Centre, Guy’s and St. Thomas NHS Foundation Trust, London, UK; ^3^Neonatal Intensive Care Centre, King’s College Hospital NHS Foundation Trust, London, UK

**Keywords:** bronchopulmonary dysplasia, prematurity, resistance, compliance, diuretics, bronchodilators, corticosteroids

## Abstract

Bronchopulmonary dysplasia (BPD) is a chronic lung disease that predominantly affects prematurely born infants. Initially, BPD was described in infants who had suffered severe respiratory failure and required high pressure, mechanical ventilation with high concentrations of supplementary oxygen. Now, it also occurs in very prematurely born infants who initially had minimal or even no signs of lung disease. These differences impact the nature of the lung function abnormalities suffered by “BPD” infants, which are also influenced by the criteria used to diagnose BPD and the oxygen saturation level used to determine the supplementary oxygen requirement. Key also to interpreting lung function data in this population is whether appropriate lung function tests have been used and in an adequately sized population to make meaningful conclusions. It should also be emphasized that BPD is a poor predictor of long-term respiratory morbidity. Bearing in mind those caveats, studies have consistently demonstrated that infants who develop BPD have low compliance and functional residual capacities and raised resistances in the neonatal period. There is, however, no agreement with regard to which early lung function measurement predicts the development of BPD, likely reflecting different techniques were used in different populations in often underpowered studies. During infancy, lung function generally improves, but importantly airflow limitation persists and small airway function appears to decline. Improvements in lung function following administration of diuretics or bronchodilators have not translated into long-term improvements in respiratory outcomes. By contrast, early differences in lung function related to different ventilation modes have led to investigation and demonstration that prophylactic, neonatal high-frequency oscillation appears to protect small airway function.

## Introduction

Bronchopulmonary dysplasia (BPD) is a chronic lung disease that predominantly affects prematurely born infants, but can occur in those born at term if they are subjected to high-inflation pressures. Initially, BPD was described in prematurely born infants who often had suffered severe respiratory failure and required high pressure, mechanical ventilation with high concentrations of supplementary oxygen, often coined old BPD. Such infants were not routinely exposed to either antenatal corticosteroids or postnatal surfactant. BPD now also occurs in very prematurely born infants who initially had minimal or even no signs of lung disease, the so-called new BPD (1). At postmortem, infants with new rather than old BPD have less interstitial fibrosis, but an arrest in acinar development, resulting in fewer and larger alveoli (2). The chest radiograph (CXR) appearance of new BPD, that is small volume, hazy lung fields, is very different from the cystic abnormalities and interstitial fibrosis seen in “old” BPD. As a consequence, lung function abnormalities are likely to differ according to whether an infant is developing old or new BPD. The nature of the lung function abnormalities may also be influenced by the criteria used to diagnose BPD. These have included oxygen dependency at 28 days or 36 weeks post conceptional age (PCA) with or without radiological abnormalities. Nowadays, there is a consensus that infants should be diagnosed as having BPD if infants are oxygen dependent at 28 days after birth (3). They are then classified as suffering from mild, moderate, or severe BPD according to their respiratory support requirement at a later date (36 weeks PCA if born prematurely) (3) (Table 1). A further problem is that different levels of oxygen saturation have been used to determine the need for supplementary oxygen leading to wide variations in the occurrence of BPD (4). As a consequence, it is now recommended that an oxygen reduction test is used to determine whether supplementary oxygen is still required (5). Of note, lung function abnormalities as assessed by pulmonary function testing are not part of the current criteria to diagnose BPD, likely reflecting pulmonary function testing is not routinely available in all neonatal intensive care units (NICU).

**Table 1:** **BPD severity modified from Jobe and Bancalari (3)**.

Infants <32 weeks of gestational age are assessed at 36 weeks PCA or at discharge home, whichever came first
Infants born at 32 weeks of gestation or greater are assessed at 56 days postnatal age or discharge home, whichever came first
The severity of BPD being graded in both groups accordingly
• mild BPD – breathing air
• moderate BPD – requirement for <30% supplementary oxygen
• severe BPD – requirement for more that 30% oxygen and/or positive pressure ventilation or nasal continuous positive airway pressure (CPAP)

Bearing in mind those caveats, an aim of this review is to describe lung function abnormalities in infants developing or with established BPD and how they change with increasing postnatal age during infancy. In addition, we will highlight if lung function testing in the NICU and during infancy gives added value. For example, do lung function test results so accurately predict BPD development that they could be used to identify infants who would benefit from intervention strategies or have improvements in lung function given an early indication of clinically efficacious interventions.

It, however, should be emphasized at the outset that a diagnosis of BPD is a poor predictor of ongoing pulmonary problems (6) and infants with and without BPD suffer respiratory morbidity at follow-up. In addition, certain randomized controlled trials (RCTs), which have demonstrated a reduction in BPD, have not been associated with improvements in long-term respiratory outcome (7) and equally interventions influencing long-term respiratory morbidity were not associated with a reduction in BPD (8, 9).

## Appropriate Lung Function Tests

Key to interpreting lung function data is whether an appropriate test has been used and whether the test has been applied robustly. In infants with evolving or established BPD, the techniques have strengths and weaknesses. Dynamic lung compliance measurements do not require airway occlusions, which may be poorly tolerated in infants with respiratory distress. Esophageal pressure measurements, however, are required, which may not accurately reflect pleural pressure changes in prematurely born infants who have a floppy chest wall or in the presence of lung disease. Single breath mechanics do require airway occlusions and the underlying assumptions are invalid if the respiratory system cannot be described as single-compartment model. The high resistance of small endotracheal tubes (10) may invalidate attempts to detect small changes in resistance in intubated infants. Assessments of functional residual capacity (FRC) by gas dilution or washout, can be applied in ventilated infants, but may underestimate the FRC if insufficient time is allowed for complete equilibration (11). Nitrogen washout with pure oxygen is impractical for ventilated infants receiving a high-fractional inspired oxygen concentration and inappropriate for infants at risk of retinopathy of prematurity. Inert gases such as helium and sulfur hexafluoride (SF6) avoid these problems. As a relatively heavy gas with low diffusivity, SF6 has the additional advantage of being less susceptible than helium to leaks (12), especially those occurring around an uncuffed endotracheal tube. Certain centers, however, use shouldered tubes, which have been demonstrated to have minimal or no leak (13). The major strength of measuring lung volume using infant whole-body plethysmography is that the total lung volume can be measured and hence, if used in conjunction with a gas dilution technique, can provide an assessment of hyperinflation and gas trapping (14). Systems are commercially available, but depend on electronic manipulation to close the pressure flow loop, which can result in erroneous results (15). A further disadvantage is that plethysmographs are not suitable for cot side measurements. In addition, the accuracy of plethysmographic measurements is dependent on rapid equilibration of pressures during respiratory efforts against occlusions, so that pressure changes at the airway opening reflect those in the alveoli (16). In the presence of severe airway obstruction, this may not occur, resulting in a phase lag between airway pressure and box volume, usually resulting in overestimation of lung volume (11). As infants actively elevate their end expiratory level, all lung volume measurements should be made during quiet, non-rapid eye movement sleep (17). Respiratory impedance plethysmography (RIP) can provide information on respiratory rate and the degree of thoracoabdominal asynchrony. The interpretation of the results is dependent on sleep state and volume calibration is not possible in this population.

## Lung Function Abnormalities

Among infants with “old” BPD, increased resistance in the first week after birth and increased total respiratory and expiratory resistance with severe flow limitation, especially at low lung volumes at 28 days after birth was reported (18). More recent studies, which have included infants who usually have received surfactant, have demonstrated somewhat differing results. In a group of infants, the majority of whom had been given rescue surfactant, respiratory system resistance (Rrs) was abnormal at 10 days after birth, but then there was progressive improvement to normal values (19). In a series in which infants were given prophylactic surfactant and exposed to antenatal steroids, Rrs differed significantly between those who did and did not develop BPD on day three, but not at 14 days after birth (20).

Compliance is initially low in infants destined to develop BPD. In one series of ventilated infants, compliance of the respiratory system (Crs), using the single breath technique, was 50% of predicted at 10 days of age (19). Interestingly, there was a positive correlation (*r* = 0.8, *p* < 0.001) between those Crs results and maximal flow at FRC ($V_{\max}^{\prime }$ FRC) using the forced expiratory volume technique at 2 years of age. In the presence of low-saccular compliance, the highly compliant distal bronchial tree is preferentially over distended resulting in marked distortion of both distal and central areas during mechanical ventilation (21). The authors (19), therefore, postulated that in infants with very low lung compliance in the neonatal period, cyclic bronchiolar stretching during positive pressure ventilation resulted in terminal airway ischemia and necrosis and subsequent fibrosis and smooth muscle hypertrophy. Compliance and lung volume abnormalities may persist over the neonatal period. Comparison of FRC results from 16 BPD infants (oxygen dependent for more than 28 days) and 8 infants without BPD demonstrated the BPD group had lower FRCs at both 14 and 28 days (22). Similarly, serial measurements of Crs and FRC in 74 infants, median gestational age 30 weeks, 35 of whom developed BPD (23 had moderate/severe BPD) demonstrated that those developing BPD, particularly moderate/severe BPD had significantly lower Crs and FRC results throughout the neonatal period compared to those who did not develop BPD (20). CXR thoracic areas and FRC measurements assessed in the first 72 h after birth in 53 infants, median gestational age of 28 weeks, also demonstrated lower FRCs in the BPD group, but the CXR thoracic areas were higher in the infants who subsequently developed BPD (oxygen dependency at 28 days) perhaps indicating gas trapping. The differences were particularly marked in infants who developed moderate/severe BPD (23). The reduced Crs in the neonatal period may be due to ongoing surfactant abnormalities, edema, and atelectasis. Similarly, the initial functional lung volume is likely reduced because of atelectasis.

During evolving BPD, there is gas trapping. FRC measured by plethysmography (FRCpleth) has been reported to be elevated in infants with BPD (24, 25) and in an early study FRCpleth was higher than FRC assessed by nitrogen washout (26). In established BPD, functional lung volume around term equivalent has been reported to still be significantly reduced compared, to data, from healthy term born infants both in an early (27) and in a more recent (24) study and associated with disturbed gas mixing (28). Those results appear pertinent to the present population of prematurely born infants who develop BPD. In a subsequent study (29), approximately 50% of the infants were exposed to antenatal steroids and 100% of those who developed moderate or severe BPD received surfactant. At term corrected, the severe BPD group had lower FRC, less efficient gas mixing, and higher specific conductance than those with mild and moderate BPD or the prematurely born controls. The infants with mild or moderate BPD infants also differed from the controls (29). BPD infants have also been shown to have significant increases in FRC, residual volume (RV), and RV/total lung capacity, which were more marked in those with recurrent wheeze, suggestive of hyperinflation and air-trapping (24). In a follow-up of prematurely born infants all born at <29 weeks of gestational age, two-thirds of whom had BPD, the degree of gas trapping significantly correlated with days of wheeze (30).

Results from a small study suggest that single photon emission computed tomography (SPECT) may provide additional information about regional lung function in BPD infants (31). SPECT was used to measure the distribution of lung ventilation (V) and perfusion (Q) in 30 BPD infants at a median PCA of 37 weeks. An unsatisfactory V/Q match was not correlated with the time spent on supplemental oxygen or CPAP, but was significantly negatively correlated with the time spent on mechanical ventilation. Increasing severity of BPD, however, was not consistently associated with the degree of V/Q mismatch.

Other lung function abnormalities in infancy suggest impairment of alveolar development after very premature birth. Pulmonary diffusing capacity and alveolar volume were assessed at 11.6 months of age using a single breath hold maneuver at elevated lung volume in 39 BPD infants (oxygen requirement at 36 weeks) and 61 term born controls. The BPD patients had reduced pulmonary diffusing capacity when adjusted for body length or alveolar volume (32).

## Longitudinal Assessment

Early studies assessing serial lung function highlighted that lung compliance and FRC improved with increasing age (19, 27), such that by 2 years of age they had reached the normal range (19). In addition, during the first 2 years after birth, a relative increase in FRC using a gas dilution technique was reported (19). More recently, results were apparently at variance as assessment of 55 sedated VLBW infants (29 with BPD, oxygen dependency at 36 weeks PCA) at 50, 70, and 100 weeks of PCA demonstrated significantly lower tidal volume, minute ventilation, compliance, and FRC results in the BPD infants (33). Those differences, however, were no longer statistically significant once the results were normalized for body weight, which was significantly lower in the BPD group (33).

Airflow limitation, however, appears to be a persisting problem. An early report highlighted that lower airway obstruction persisted in infants with BPD who had severe disease as indicated by requirement for a tracheostomy (34). Longitudinal assessment demonstrated that, in infants with severe BPD, abnormalities in forced vital capacity (FVC) took longer to improve them eventually reaching the normal range by 3 years of age, but there was no improvement in forced expiratory flow at 75% of vital capacity (FEF_75_) over the study period (34). In another study, 70% of BPD infants assessed at 2 years had low flow rates below 40% of that predicted, whereas lung volumes, Crs, and Rrs results were in the normal range (19). More recent results in the present population of prematurely born infants confirm those results (25, 34). Assessment of 44 children with BPD (oxygen dependency at 28 days or 36 weeks PCA with CXR changes) highlighted that there were no improvements in $V_{\max}^{\prime }$ FRC at 6, 12, and 24 months (35). In a longitudinal study, which examined infants at a PCA of 58 weeks and then 33 weeks later, the group mean lung volumes and flows tracked at or near their previous values, that is, there was a lack of catch up growth. There were, however, improvements in lung function in those with above average growth (25).

Serial lung function measurements in infants with BPD have shown a decline in small airway function (as evidenced by assessments of $V_{\max}^{\prime }$ FRC) during the first year after birth (36). Similar changes in small airway function, however, have been reported in healthy, unsedated, prematurely born infants (37). Those findings emphasize the importance of using an appropriate control group when interpreting long-term effects of respiratory disease or management strategies in the neonatal period.

## Prediction of BPD

Initial studies focused on compliance and resistance results with differing results. In certain studies, resistance, but not compliance, results were predictive of BPD development. One study, however, included only 20 infants who had required mechanical ventilation for at least 3 days; 8 developed BPD (supplementary oxygen for longer than 28 days) (38). In a study of 46 infants with a birth weight <1.0 kg, those who subsequently developed BPD had significantly higher Rrs, but not Crs, at 1 week and Rrs was significantly higher in those with evolving BPD throughout the neonatal period (39). In another study, Rrs but not Crs before surfactant therapy was associated with an increased risk of BPD. Areas under receiver operator characteristic (ROC) curves were reported and demonstrated that Rrs performed similarly to gestational age and birth weight (40).

By contrast, other studies have demonstrated compliance rather than resistance was predictive of BPD development. Dynamic compliance, assessed using flow measurements from a pneumotachograph related to mean airway pressures in 47 infants mean gestational age of 30 weeks in the first 3 days after birth, was significantly lower in those who developed BPD, which was diagnosed using radiological criteria (41). In a subsequent study, an occlusion technique was used to determine appropriate esophageal balloon placement and longitudinal assessment over the first month was made on 143 infants with a gestational age of 27–30 weeks. The model, which included gestational age and dynamic pulmonary compliance, had the highest positive predictive accuracy (100%) for BPD (the need for supplemental oxygen at 4 weeks of age), whereas the predictive value of total pulmonary resistance was minimal (42). In 39 ventilated infants with a mean gestational age 26–28 weeks, the predictive ability of the results of the interrupter technique was compared to respiratory mechanics results obtained during mechanical ventilation. Dynamic compliance of the respiratory system on day 1, birth weight and gestational age were all significantly lower in the BPD infants (BPD diagnosed if the infant developed lung disease in the first week after birth, was oxygen dependent at 28 days of age and developed characteristic chest x-ray changes), but there were no significant differences in the interrupter technique results. Dynamic compliance of the respiratory system was a better independent predictor of BPD development than gestational age or birth weight (43). More recently, among 52 prematurely born infants who were ventilated for more than 72 h and had received a single dose of porcine surfactant, initial compliance results did not differ between infants who went on to develop mild or severe BPD, but at days 7 and 10 the “severe” group had significantly poorer compliance results. BPD was diagnosed as a requirement of supplementary oxygen at 28 days to maintain oxygen saturation above 95%; the severity of BPD was determined by the CXR score. Compliance but not resistance on days 7 and 10 were predictive of severe BPD (44).

In other studies, neither compliance nor resistance results were predictive of BPD development (45, 46). In a study of 104 ventilator-dependent infants, with a mean gestational age 27–30 weeks, respiratory mechanics were measured using an airway occlusion technique between 6 and 48 h after birth and corrected for body length. Birth weight, but not respiratory system mechanics, predicted BPD development (supplemental oxygen requirement 28 days after birth) (45). In a series of 58 infants who had RDS, compliance and resistance results were assessed using a commercially available system. BPD was diagnosed as oxygen supplementation, respiratory distress, and an abnormal CXR at 28 days. Neither lung compliance nor pulmonary resistance on days 1–4 predicted BPD, but gestational age and a ventilatory index on day 3 (ventilator frequency × maximum inspiratory pressure) were predictive (46).

It is not possible to conclude from the above studies whether assessment of lung mechanics is helpful in predicting BPD development. The discrepancy in the results likely reflects that different techniques were used in different populations. Few of the above studies included a sample size calculation and thus there may have been both type I and II errors.

More recent studies have investigated whether assessment of FRC might be predictive of BPD. In 100 infants with a median gestational age 28 weeks and ventilated within 6 h of birth, FRCHe ≥19 ml/kg and a low gestational age in the first 48 h were more accurate predictors of BPD at 28 days than Crs or Rrs. Indeed, if only the 50 infants whose gestational age was ≤28 weeks of gestation were considered, a low FRC on day 2 was the best predictor of BPD development (47). Subsequently, the results of FRC and Crs on days 3 and 14 after birth were compared to a marker of inflammation, end-tidal carbon monoxide (ETCO). Seventy-eight infants with a median gestational age of 29 weeks were assessed; 39 developed BPD (oxygen dependency at 28 days); a sample size calculation was given. Gestational age, birth weight, ETCO, FRC, and Crs results on days 3 and 14 differed significantly between those who did and did not develop BPD. Multifactorial logistic analysis, however, demonstrated only birth weight and ETCO levels on day 14 were significant predictors of BPD with an area under the ROC curve of 0.97. Those ETCO results indicate ongoing inflammation in infants developing BPD (48).

## Response to Therapies

Lung function tests have been used to assess the response to therapies in infants with evolving or established BPD. Variable and often conflicting results have been reported, which reflects the use of different techniques, some of which were inappropriate in this age group, lack of a sample size, or an inappropriate sample size based on too optimistic a view of the likely effect. A further problem in interpreting the results are that many of the studies were reported more than 20 years ago and, therefore, include infants not exposed to antenatal steroids or postnatal surfactant, which could have affected their response to the intervention. More importantly, reported changes in lung function results were not always accompanied by a change in clinical status or affected longer-term outcome.

### Diuretics

Bronchopulmonary dysplasia infants often are poorly tolerant of fluid loads with excessive weight gain on standard fluid regimens. As a consequence, diuretics are frequently prescribed but have short- and long-term side-effects including electrolyte disturbance and nephrocalcinosis. It is, therefore, important to determine if they are having a positive effect. Early results demonstrated that administration of frusemide acutely increased lung compliance and reduced airway resistance (49, 50) was associated with a reduction in ventilator requirements (51) and transient improvements in blood gases (49). A systematic review, however, demonstrated that in prematurely born infants <3 weeks of age, frusemide administration had either inconsistent or no detectable effects (52). In addition, in 16 spontaneously breathing infants with postnatal ages ranging from 4 to 35 weeks, a single dose of frusemide was associated with improvement in pulmonary compliance but not blood gases or resistance. Furthermore, a 6- to 10-day course was associated with improvement in compliance and resistance (53), but better oxygenation was only achieved in 6 of the 16 infants.

Nebulized frusemide has been given with the hope that this would improve respiratory function while avoiding the systemic complications. In a study of eight ventilated infants with a mean postnatal age of 33 days, the effects of 0.1, 0.25, 0.5, and 1.0 mg/kg of nebulized frusemide were assessed. A dose of 1 mg/kg was associated with a 28% improvement in pulmonary resistance and a 51% improvement in pulmonary compliance at 1 h; the effects lasted for least 4 h. A systematic review demonstrated a significant improvement in tidal volume after 1 and 2 h, but no improvement in compliance at either time point (54).

Due to the side-effects of frusemide, infants who require chronic diuretic therapy are often changed to chlorothiazide and spironolactone. It had been suggested that the combination improved the outcome of babies with severe BPD (55). In a randomized, double blind, crossover trial, the effects of oral diuretics (chlorothiazide 20 mg/kg/dose and spironolactone 1.5 mg/kg/dose) given twice daily for a week were compared to placebo (56). The mean airway resistance, specific airway conductance, and dynamic compliance improved significantly, but only 10 infants were included in the study. In a further randomized trial (57), spironolactone and chlorothiazide were compared to placebo in 43 oxygen dependent BPD infants. Infants in the treatment group only had improvements in dynamic pulmonary compliance and airway resistance and, at 4 weeks after study entry, required less supplementary oxygen than the placebo group. There were, however, no significant differences in the pulmonary function test results after discontinuation of treatment, nor in the total number of days supplementary oxygen was required between the two groups. Orally administered diuretics (chlorothiazide and spironolactone) in combination with theophylline have been demonstrated to have an additive positive effect on dynamic compliance, but no effect on clinical outcomes were reported (58).

### Bronchodilators

Inhaled bronchodilators have been reported to improve pulmonary resistance, dynamic compliance, and transcutaneous blood gases when administered to ventilated babies with BPD at approximately 1 month of age (59) and reduce airway resistance in infants with BPD at term (60–62). Intravenously administered salbutamol (30 μg/kg) in six infants aged between 54 and 105 days resulted in an improvement in respiratory system compliance and resistance using the occlusion technique, but there was no correlation between salbutamol serum concentration and pulmonary function changes (63). Comparison of the effectiveness of aerosol and intravenous delivery of salbutamol was made in eight ventilator-dependent infants in randomized order; there were similar improvements in pulmonary mechanics with the two delivery methods (64). In a comparison of different inhalation devices in infants during unassisted breathing as well as in a group of ventilated infants (65, 66), it was reported that both Crs and Rrs were sensitive indicators of a bronchodilator effect. Interpretation of those data is, however, limited because the studies were performed prior to standardization of the technique and intra-individual variability was not reported.

Synergism was reported between ipratropium bromide (IB) and salbutamol in improving pulmonary mechanics in ventilated infants for up to 1–2 h after administration (67). In 10, ventilator-dependent infants, mean age 25 days, various dose of IB (75, 125, and 175 μg) plus 0.04 mg salbutamol were compared to placebo. Rrs and Crs were measured by the single breath occlusion technique. The greatest decline in Rrs (mean 26%) was seen after 175 μg IB with salbutamol. The authors, therefore, concluded that muscarinic receptors contribute to the increased bronchomotor tone of infants with BPD (67). No synergy, however, was shown between metaproterenol and atropine with lung function returning to baseline after both treatments (68).

All of the above studies were undertaken more than 20 years ago, there were no sample size calculations and no long-term benefits reported. As a consequence, in the present population of premature infants, diuretics should only be given to treat incipient heart failure in infants with evolving or established BPD and stopped as soon as that problem ceases. Equally, bronchodilators should only be administered to treat troublesome wheeze and continued if the administration is associated with a reduction in respiratory support requirements. Administration should be via a metered dose inhaler and spacer rather than a nebulizer, as the nebulizing fluid can cause bronchoconstriction (69).

### Dexamethasone

The efficacy of dexamethasone to prevent and treat BPD has been tested in many RCTs. Unfortunately, although systemically administered steroids have many positive effects, they have short- and long-term adverse effects. As a consequence, attention has been given to assessing the response to lower doses and inhaled steroids. A 1-week tapering course of dexamethasone starting at 0.5 mg/kg/day given at 7–14 days of age in ventilator dependent, VLBW infants increased pulmonary compliance and decreased the incidence of BPD at 36 weeks PCA (70). In a subsequent study, the effectiveness of that dose (total dose 2.35 mg/kg) compared to a lower dose (total dose 1 mg/kg) was compared. FRC using a nitrogen washout technique and Crs by an occlusion technique were measured in infants at a mean age of 11 days. The sample size was powered to detect that the increase in FRC in the lower dose group would be more than 10% smaller than in the higher dose group. No significant differences were shown (71). In a RCT, 10 days of dexamethasone were compared to 100 μg qds per day of budesonide in 40 infants with a median postnatal age 27 days. The study was powered to detect a difference of 7% in the inspired oxygen requirement 1 week after starting therapy. After 36 h, only the systemic group had significant reductions in the inspired oxygen concentration and Crs and at 1 week the systemic group had significantly better results than the inhaled group (72).

### Respiratory support

Lung function testing has been used to compare the acute and longer-term efficacy of respiratory support techniques. For example, in a randomized crossover study, proportional assist ventilation (PAV) and assist control ventilation (ACV) were examined in infants with evolving BPD. When on PAV the infants had superior respiratory muscle strength and a lower work of breathing and this was associated with better oxygenation (73). Follow-up studies assessing the efficacy of ventilation modes demonstrate the importance of which lung function technique was employed. In a follow-up study of an RCT, no advantage of HFO over conventional ventilation (CMV) was reported with respect to lung function at 1 year corrected, but small airway function was only assessed by evaluation of gas trapping (74). In another study, $V_{\max}^{\prime }$ FRC was assessed at 6 and 12 months in infants who developed BPD. Those who were initially supported by CMV showed the expected decline in small airway function, but this was not seen in those seen in the HRO group. As a consequence, at 12 months, the HFO group had superior lung function (75). The results of that non-randomized study suggested that HFO might protect small airway function. Follow-up at 11–14 years of children who had been entered into a neonatal RCT of prophylactic HFO has subsequently proved that hypothesis (9).

## Conclusion and Future Directions

- Infants who develop BPD have low compliance and lung volumes and elevated resistances in the neonatal period.
- During infancy, lung function generally improves, but airflow limitation persists and small airway function declines.
- Improvements in lung function following administration of diuretics or bronchodilators have not translated into long-term improvements in respiratory outcomes, but assessment at follow-up has demonstrated neonatal high-frequency oscillation appears to protect small airway function.
- Further investigation should be undertaken to determine whether a lung function assessment accurately predicts chronic respiratory morbidity and hence prophylactic interventions can be appropriately targeted.
- Lung function assessment at follow-up should be incorporated into all neonatal RCTs, which are aimed at improving respiratory outcome.

## Author Contributions

Both authors undertook a literature review and produced the manuscript.

## Conflict of Interest Statement

Anne Greenough has held grants from various ventilator manufacturers; Anne Greenough has received honoraria for giving lectures and advising various ventilator manufacturers. Anoop Pahuja has no conflict of interest to declare.
